# Noninvasive Ventilation in Patients With COVID-19-Related Acute Hypoxemic Respiratory Failure: A Retrospective Cohort Study

**DOI:** 10.3389/fmed.2021.638201

**Published:** 2021-05-24

**Authors:** Yingyun Fu, Lili Guan, Weibo Wu, Jing Yuan, Shanshan Zha, Junmin Wen, Zhenghao Lin, Chen Qiu, Rongchang Chen, Lei Liu

**Affiliations:** ^1^Shenzhen Institute of Respiratory Diseases, Shenzhen People's Hospital (The Second Clinical Medical College, Jinan University; The First Affiliated Hospital, South University of Science and Technology), Shenzhen, China; ^2^National Clinical Research Center for Infectious Disease, The Third People's Hospital of Shenzhen, The Second Affiliated Hospital of Southern University of Science and Technology, Shenzhen, China; ^3^Fuwai Hospital Chinese Academy of Medical Sciences, Shenzhen, China

**Keywords:** coronavirus disease 2019, noninvasive ventilation, rescue therapy, delayed intubation, acute hypoxemic respiratory failure

## Abstract

**Introduction:** Noninvasive ventilation (NIV) has been used to alleviate hypoxemia and dyspnea, but there is no consensus on the application of NIV in patients with coronavirus disease 2019 (COVID-19). Some staff use NIV as the rescue therapy which might lead to the adverse outcomes. This study was to identify early factors associated with intubation to help the medical staff select appropriate patients for receiving NIV treatment.

**Methods:** Patients with laboratory-confirmed COVID-19 who were treated with NIV in emergency department or ICU of the Third People's Hospital (the only designated hospital for treating COVID-19 in Shenzhen) between January 1 and August 31, 2020, were retrospectively analyzed.

**Results:** Thirty-nine patients with COVID-19 treated with NIV were included; of them, 16 (41%) received endotracheal intubation and 3 (8%) died. Significant differences were observed between intubated and non-intubated patients in PaO_2_/FiO_2_ before NIV initiation, hospitalization duration, NIV as the rescue therapy, and PaO_2_/FiO_2_ of ≤200 mmHg after 1–2 h of NIV initiation. Notably, 1–2 h after NIV initiation, a PaO_2_/FiO_2_ of ≤200 mmHg (odds ratio [OR], 9.35; 95% confidence interval [CI], 1.84–47.62; *P* = 0.007) and NIV as the rescue therapy (OR, 5.43; 95% CI, 1.09–27.12; *P* = 0.039) were the risk factors for intubation.

**Conclusions:** In patients with COVID-19-related acute hypoxemic respiratory failure receiving NIV, close attention should be paid to PaO_2_/FiO_2_ after 1–2 h of NIV initiation. Also, using NIV as rescue therapy should draw our awareness that it might delay escalation of respiratory support and lead to adverse outcomes.

## Introduction

Since the identification of an initial cluster of patients in December 2019, the coronavirus disease 2019 (COVID-19) pandemic has continued to wreak global havoc. So far, ~20% of patients have been categorized as severely or critically ill, presenting with acute hypoxemic respiratory failure (AHRF) (1). Medical resources have been in short supply owing to the vast number of patients. Moreover, early intubation has inevitably led to some complications in these patients (2).

Using noninvasive respiratory support to treat patients without intubation can save medical resources and reduce the incidence of pain and complications (3–5). Noninvasive ventilation (NIV) has been used to alleviate hypoxemia and dyspnea in patients with COVID-19 during the pandemic (6). However, using NIV to treat AHRF remains showing some discrepancies, probably due to the patient selection and NIV parameter settings (7, 8). For now, there is no unified consensus on the application of NIV in patients with COVID-19-related AHRF; most of the consensus are based on previous experience in the treatment of viral pneumonia (9, 10). Medical staff tend to base their choice on personal preference or experience because there is no evidence-based recommendation for the NIV selection (6, 11). In addition, some staff tend to use NIV as the rescue therapy after conventional oxygen therapy or high-flow nasal oxygen therapy (HFNO) had failed, which might delay the escalation of respiratory support and lead to the adverse outcomes.

Therefore, in this retrospective cohort study, we aimed to identify early factors associated with intubation to help the medical staff select appropriate patients for receiving NIV treatment and avoid delayed intubation.

## Methods

**Figure 1:**
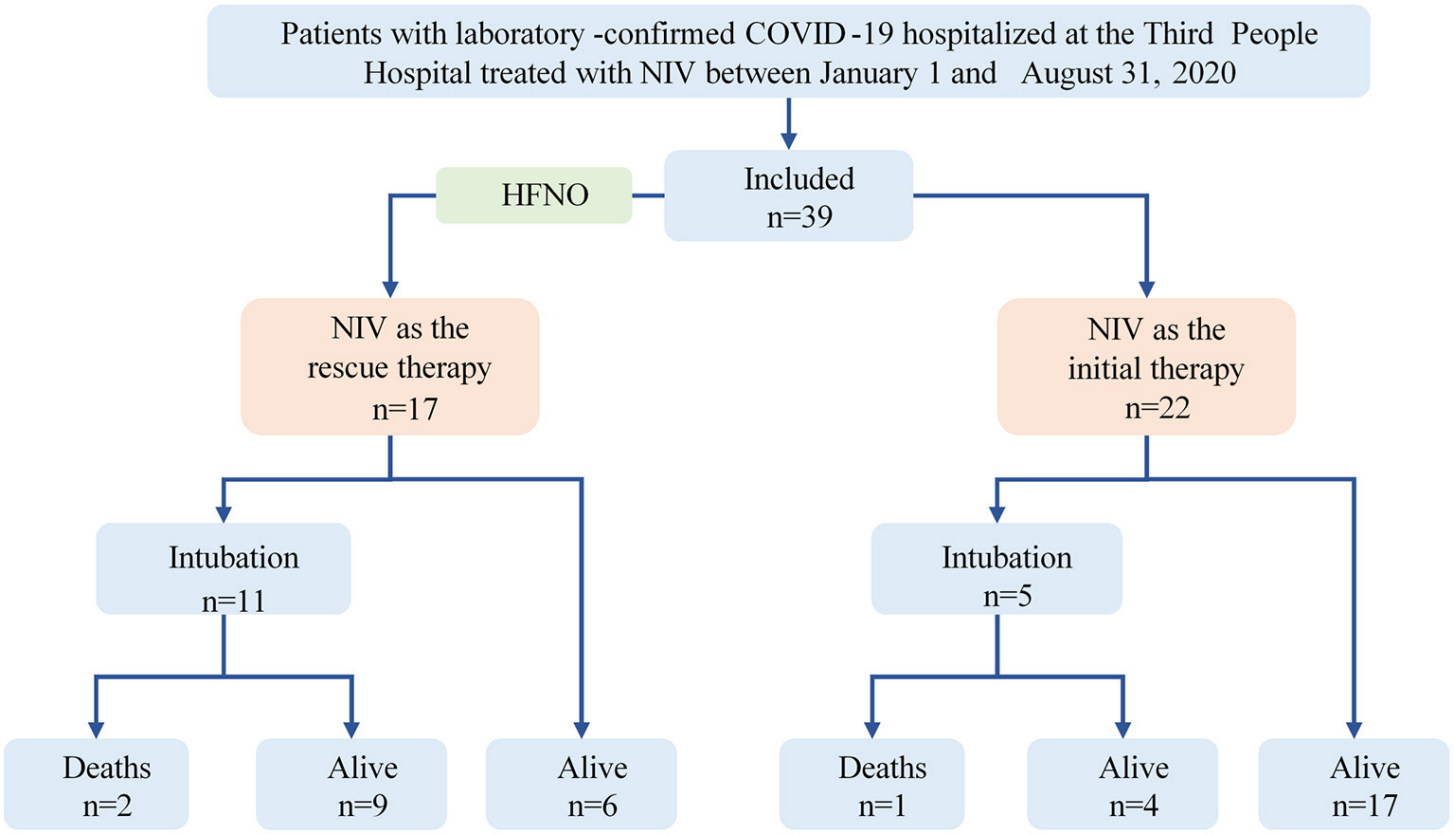
Flowchart of the study. COVID-19, coronavirus disease 2019; NIV, noninvasive ventilation; HFNO, high-flow nasal oxygen therapy.

In this retrospective cohort study, patients with laboratory-confirmed COVID-19 in the emergency department or ICU of the Third People's Hospital which is the only designated hospital for treating COVID-19 in Shenzhen, a megacity with a population of more than 10 million, between January 1 and August 31, 2020, were included (Figure 1). All patients who were treated with NIV during their hospital stay and were identified from electronic medical records, were included. The treatment regimen was in accordance with the Chinese COVID-2019 treatment guidelines (12), and all patients have already been discharged from the hospital, except three deaths.

Demographic and clinical data and information on ventilatory settings and arterial blood gas samples were collected before and 1–2 h after NIV initiation. Two clinicians independently gathered demographic and clinical data from electronic medical records using standardized data collection forms; any variances or discrepancies were discussed, and a third clinician moderated and adjudicated the validity of disputed data.

NIV as the initial treatment was defined as using NIV as the first choice to correct hypoxemia after using conventional oxygen therapy. NIV as the rescue therapy was defined as using NIV to treat patients with AHRF in whom treatment with HFNO had failed. The criteria for intubation included the signs of persisting or worsening respiratory failure, hemodynamic instability, or consciousness deterioration (12). The primary outcome of the study was to identify early factors associated with intubation, whereas secondary outcomes included hospitalization duration and all-cause mortality during hospitalization.

SPSS version 19.0 (IBM SPSS Statistics, Armonk, NY, USA) was used for data analysis. Data were presented as median (interquartile range [IQR]), or number (percentage) as appropriate. For continuous variables, comparisons were performed using Student's *t*-test for normally distributed data and nonparametric test for non-normally distributed data. For categorical variables, Pearson's chi-square test or Fisher's exact test was used. Factors associated with intubation were assessed via backward multivariate logistic regression analyses. The Kaplan–Meier method was used to evaluate the 28th day intubation rate from hospital admission and symptom onset, and between-group differences were estimated using the log-rank test. A two-tailed *P* < 0.05 was considered significant. The ethics committee of the Third People's Hospital approved this study, and the trial was registered with www.chictr.org.cn (ChiCTR2000039567).

## Results

In total, among 423 hospitalized patients with COVID-19 in Shenzhen, 39 patients with COVID-19 (9%) who were treated with NIV were included in the study; of them, 16 (41%) received endotracheal intubation, and 3 (8%) died. All patients received bilevel ventilation. The median initial inspiratory positive airway pressure and expiratory positive airway pressure were 13.0 (IQR, 12.0–14.0) cmH_2_O and 6.0 (IQR, 6.0–6.5) cmH_2_O, respectively, with a median fractional inspired oxygen (FiO_2_) of 0.5 (IQR, 0.4–0.5).

There is no significant difference between intubated and non-intubated patients in the baseline demographic, comorbidities, severity blood markers (white blood cell count, lymphocyte count, procalcitonin, d-dimer) and severity scores (APACHE II score) (Table 1). Patients who required intubation had lower arterial oxygen partial pressure (PaO_2_) to FiO_2_ ratio (PaO_2_/FiO_2_) before receiving NIV, higher proportion of using NIV as the rescue therapy, and longer hospitalization duration (Table 1). Significant differences were observed between intubated and non-intubated patients in PaO_2_/FiO_2_ before NIV initiation (144.1 [IQR, 126.0–169.5] mmHg vs. 180.0 [IQR, 151.4–226.5] mmHg; *P* = 0.016), hospitalization duration (46.0 [IQR, 35.0–52.5] days vs. 34.0 [IQR, 28.0–40.0] days; *P* = 0.007), NIV as the rescue therapy (68.8 vs. 26.1%; *P* = 0.011), PaO_2_/FiO_2_ of ≤200 mmHg after 1–2 h of NIV initiation (68.8 vs. 17.4%; *P* = 0.002), and the pressure support level (6.0 [IQR, 6.0–6.3] cmH_2_O vs. 7.0 [IQR, 6.0–7.0] cmH_2_O; *P* = 0.046). Furthermore, a trend of higher in-hospital mortality was observed in the intubated patients (18.8 vs. 0%; *P* = 0.061). Multivariate logistic regression analysis showed that 1–2 h after NIV initiation, a PaO_2_/FiO_2_ of ≤200 mmHg (odds ratio [OR], 9.35; 95% confidence interval [CI], 1.84–47.62; *P* = 0.007) and NIV as the rescue therapy (OR, 5.43; 95% CI, 1.09–27.12; *P* = 0.039) were the risk factors for intubation (Table 1).

**Table 1:** Comparison of baseline clinical characteristics, NIV therapy, and clinical outcomes in patients who required intubation or not intubation.

	**Intubation (*n* = 16)**	**Nonintubation (*n* = 23)**	**Unadjusted *p*-value**	**OR (95% CI)**	**Adjusted *p*-value**
**Baseline clinical characteristics**					
Age, years	65.0 (58.5–69.0)	62.0 (59.5–65.0)	0.343		
Male, *n* (%)	11 (68.8)	15 (65.2)	0.818		
BMI, kg/m^2^	24.6 (21.7–26.2)	25.4 (22.8–27.1)	0.394		
White blood cell count, ×10^9^/L	5.5 (4.1–6.9)	4.3 (3.5–5.1)	0.112		
Lymphocyte count, ×10^9^/L	1.0 (0.9–1.3)	1.1 (0.8–1.4)	0.746		
Procalcitonin, ng/ml	0.08 (0.06–0.11)	0.07 (0.05–0.10)	0.563		
D-dimer, μg/ml	0.7 (0.5–1.2)	0.6 (0.4–0.8)	0.107		
**Comorbidities**					
Chronic respiratory disease, *n* (%)	2 (12.5)	1 (4.3)	0.557		
Hypertension, *n* (%)	7 (43.8)	7 (30.4)	0.503		
Chronic cardiovascular disease, *n* (%)	3 (3.3)	5 (4.7)	0.820		
Chronic kidney disease, *n* (%)	0 (0)	1 (4.3)	0.398		
Chronic hepatic disease, *n* (%)	1 (6.3)	1 (4.3)	0.791		
Diabetes, *n* (%)	3 (18.8)	4 (17.4)	0.913		
Cancer, *n* (%)	1 (6.3)	1 (4.3)	0.791		
**Clinical data before NIV therapy**					
Respiratory rate, breaths/min	25.0 (24.8–26.5)	23.0 (22.0–28.5)	0.159		
Heart rate, breaths/min	83.0 (77.0–88.0)	81.0 (74.0–89.0)	0.938		
pH	7.46 (7.45–7.48)	7.45 (7.43–7.47)	0.534		
PaO_2_, mmHg	72.1 (64.6–78.8)	72.2 (63.5–80.5)	0.855		
PaO_2_/FiO_2_, mmHg	144.1 (126.0–169.5)	180.0 (151.4–226.5)	0.016		
The time interval from symptom onset to initiating NIV, days	10.5 (5.8–13.5)	11.0 (9.0–13.5)	0.489		
NIV as the rescue therapy, *n* (%)	11 (68.8)	6 (26.1)	0.011	5.43 (1.09–27.12)	0.039
APACHE II	12.0 (10.8–13.5)	10.0 (8.5–12.0)	0.094		
**Clinical data after 1**–**2 h NIV therapy**					
Respiratory rate, breaths/min	24.0 (21.5–28.5)	22.0 (20.0–23.5)	0.143		
Heart rate, breaths/min	78.0 (73.5–85.5)	78.0 (71.0–86.0)	0.935		
Pressure support, cmH_2_O	6.0 (6.0–6.3)	7.0 (6.0–7.0)	0.046		
EPAP, cmH_2_O	6.0 (6.0–6.3)	6.0 (6.0–7.0)	0.703		
PaO_2_, mmHg	87.2 (77.6–92.8)	110.0 (99.5–125.6)	<0.001		
PaO_2_/FiO_2_, mmHg	181.3 (155.2–204.7)	265.0 (215.2–306.7)	<0.001		
PaO_2_/FiO_2_ of ≤200 mmHg after 1–2 h of NIV initiation, *n* (%)	11 (68.8)	4 (17.4)	0.002	9.35 (1.84–47.62)	0.007
**Clinical outcomes**					
Hospitalization duration, days	46.0 (35.0–52.5)	34.0 (28.0–40.0)	0.007		
In-hospital mortality, *n* (%)	3 (18.8)	0 (0)	0.061		

*Data represent as median (interquartile range) or n (%)*.

*NIV, noninvasive ventilation; OR, odds ratio; CI, confidence interval; PaO_2_, arterial oxygen partial pressure; FiO_2_, fractional inspired oxygen; APACHE, acute physiology and chronic health evaluation; EPAP, expiratory positive airway pressure*.

*Variables entered in the model of logistic regression were: using NIV as the rescue therapy, APACHE II, pressure support, PaO_2_/FiO_2_ of ≤200 mmHg after 1–2 h of NIV initiation*.

**Figure 2:**
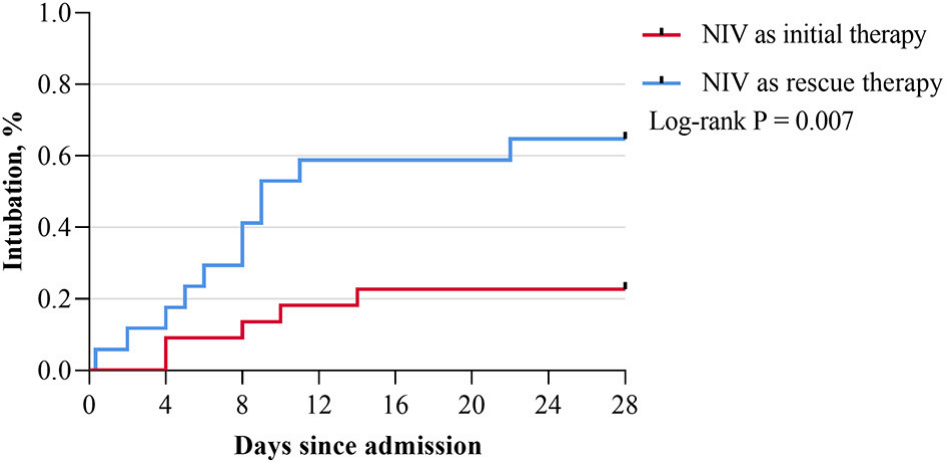
Kaplan–Meier survival curve of the intubation rate at day 28 after admission. NIV, noninvasive ventilation.

In the subgroup analysis of NIV as the rescue therapy, the medium time of using HFNO before NIV was 2 (IQR, 0.4–3.0) days, and the intubation rate at day 28 from hospital admission was much higher when comparing with NIV as the initial therapy (64.7 vs. 22.7%; *P* = 0.007; Figure 2), as well as the intubation rate from symptom onset (*P* = 0.005; Table 2). Furthermore, hospitalization duration was longer when NIV was used as the rescue therapy (42.0 [IQR, 31.5–52.5] days vs. 34.5 [IQR, 26.6–42.4] days; *P* = 0.005). There was no difference in clinical characteristics before initiating noninvasive respiratory support (HFNO or NIV), the time interval from symptom onset to initiating noninvasive respiratory support (initial therapy: 10.0 [IQR, 8.3–13.0] days vs. rescue therapy: 10.0 [IQR, 5.0–11.0] days, *P* = 0.307) and the in-hospital mortality (initial therapy: 4.5% vs. rescue therapy: 11.8%, *P* = 0.570) between patients who received NIV as initial therapy and those who received NIV as rescue therapy; however, PaO_2_/FiO_2_ was observed to be lower before initiating NIV in the latter (144.2 [IQR, 119.6–175.5] mmHg vs. 174.4 [IQR, 158.0–208.7] mmHg; *P* = 0.034) (Table 2).

**Table 2:** Comparison of baseline clinical characteristics and outcomes in patients who used NIV as initial or rescue therapy.

	**NIV as initial therapy (*n* = 22)**	**NIV as rescue therapy (*n* = 17)**	***p*-value**
Age, years	62.5 (59.3–69.0)	62.0 (59.0–66.0)	0.944
Male, *n* (%)	12 (54.5)	14 (82.4)	0.093
BMI, kg/m^2^	24.8 (22.0–27.4)	24.8 (22.9–26.6)	0.726
The time interval from symptom onset to initiating noninvasive respiratory support, days	10.0 (8.3–13.0)	10.0 (5.0–11.0)	0.307
The time interval from initiating HFNO to using NIV therapy, days	–	2 (0.4–3.0)	N.A.
**Comorbidities**			
Chronic respiratory disease, *n* (%)	0 (0)	3 (17.6)	0.074
Hypertension, *n* (%)	7 (31.8)	7 (41.2)	0.738
Chronic cardiovascular disease, *n* (%)	4 (18.2)	4 (23.5)	0.709
Chronic kidney disease, *n* (%)	0 (0)	1 (5.9)	0.436
Chronic hepatic disease, *n* (%)	1 (4.5)	1 (5.9)	0.851
Diabetes, *n* (%)	4 (18.2)	3 (17.6)	0.966
Cancer, *n* (%)	1 (4.5)	1 (5.9)	0.851
Clinical data before noninvasive respiratory support^*^			
Respiratory rate, breaths/min	24.5 (23.0–27.5)	22.0 (20.0–25.0)	0.076
Heart rate, breaths/min	85.0 (75.3–88.8)	82.0 (80.0–89.0)	0.893
pH	7.46 (7.43–7.48)	7.46 (7.45–7.47)	0.928
PaO_2_, mmHg	72.3 (61.1–79.2)	73.0 (61.5–73.8)	0.872
PaO_2_/FiO_2_, mmHg	174.4 (158.0–208.7)	179.27 (165.9–224.1)	0.468
**Clinical data before NIV therapy**			
Respiratory rate, breaths/min	24.5 (23.0–27.5)	25.0 (23.0–29.0)	0.410
Heart rate, breaths/min	85.0 (75.3–88.8)	78.0 (74.0–87.0)	0.319
pH	7.46 (7.43–7.48)	7.45 (7.43–7.47)	0.317
PaO_2_, mmHg	72.3 (61.1–79.2)	72.0 (65.3–81.0)	0.664
PaO_2_/FiO_2_, mmHg	174.4 (158.0–208.7)	144.2 (119.6–175.5)	0.034
**Clinical outcomes**			
Intubation, *n* (%)	5 (22.7)	11 (64.7)	0.007^+^
			0.005^#^
Hospitalization duration, days	35.0 (29.0–41.0)	42.0 (33.0–52.0)	0.005
In-hospital mortality, *n* (%)	1 (4.5)	2 (11.8)	0.570

*Data represent as median (interquartile range) or n (%); HFNO, high-flow nasal oxygen therapy; NIV, noninvasive ventilation; PaO_2_, arterial oxygen partial pressure; FiO_2_, fractional inspired oxygen; N.A., Not applicable*.

* *Noninvasive respiratory support refers to the HFNO or NIV*;

+ *intubation rate from hospital admission*;

# *intubation rate from symptom onset*.

## Discussion

Our findings indicate that in patients with COVID-19-related AHRF receiving NIV, a PaO_2_/FiO_2_ of ≤200 mmHg after 1–2 h of NIV initiation and using NIV as the rescue therapy are associated with a higher risk of intubation.

Recently, Franco et al. (13) performed a multicentered, retrospective study to analyze the feasibility and efficacy of using noninvasive respiratory support in patients with COVID-19-related AHRF outside ICU. All patients only used one form of noninvasive respiratory support (NIV/CPAP or HFNO) during the hospitalization. The results found that the 30-day mortality, intubation rate and length of hospitalization were similar among different noninvasive respiratory support methods, but HFNO was usually used for patients with mild COVID-19 in their clinical practice. Similar to Franco's study, we also tended to use HFNO in mild patients, but patients in our study would receive NIV as the rescue therapy when HFNO failed to improve the clinical status of patients. Actually, some medical staff in China and some consensus tended to choose HFNO as the first choice, especially during the early pandemic, because it is easy to use and has good tolerance (14). In cases where severe respiratory distress or hypoxemia could not be relieved via HFNO, NIV was used as the rescue therapy. Subsequently, the treatment failure may be related to the delayed use of NIV when using it as the rescue therapy, leading to a low PaO_2_/FiO_2_ before NIV initiation (Table 2). The rate of NIV treatment failure in our study population was comparable to those reported in two previous observational studies (41.0 vs. 44.6 vs. 49.3%) (13, 15). However, on excluding the patients using NIV as the rescue therapy in our study, the failure rate would be much lower (rescue therapy: 64.7% vs. initial therapy: 22.7%). It might imply that when using HFNO as the initial treatment for mild COVID-19 patients, patients should be closely monitored in order to avoid the delayed escalation of respiratory support. In addition, our team proposed that when considering treatment rationale, adjustable pressure, oxygen consumption, and tolerance, NIV should be considered as the first-line therapy to treat patients with mild acute respiratory distress syndrome (ARDS) (6).

Furthermore, in line with Frat's findings that 1 h after NIV initiation, a PaO_2_/FiO_2_ of ≤200 mmHg and a tidal volume of >9 mL/kg of predicted body weight are the independent predictors of intubation among patients with AHRF, we found that after initiating NIV therapy, a PaO_2_/FiO_2_ of ≤200 mmHg is an essential predictor of intubation in patients (16). We could not record the expired tidal volume because of the emergency situation during the pandemic and the retrospective nature of the study. However, studies have pointed that the pathophysiological characteristics of some patients with COVID-19 who are diagnosed with ARDS according to the Berlin definition are not entirely consistent with those of patients with typical ARDS, presenting with the mismatch of severe hypoxia and relatively good respiratory compliance (17). Therefore, whether the targeted tidal volume of patients with COVID-19-related early ARDS receiving NIV may be higher, especially among those with hypercapnia, would need more evidence in further studies (18).

Whether NIV treatment would increase the risk of viral transmission among medical staff has always been a matter of concern and debate over the past few years (19). Previous studies have shown that the maximum distance of exhaled air dissemination will be increased when using noninvasive respiratory support (20). However, there is no direct evidence indicating an increased risk of infection among medical workers while using NIV, and none of the medical staff was infected during NIV procedures performed under adequate protection in our study (20, 21). In future studies, further exploration will be needed regarding (1) the relationship between the use of NIV and the amount of virus dissemination and (2) the relationship between viral pathogenicity and the dilution effect of the increased ventilation volume.

The prone position could improve oxygenation by recruiting the collapsed region of the dorsal lung and promoting drainage of airway secretions in AHRF patients (22, 23). Recently, researchers applied prone position NIV in patients with COVID-19-related AHRF and found that it could reduce respiratory rate, improve oxygenation and comfort of patients (24). More evidence on the benefits of the prone position NIV should be investigated by comparing with standard NIV and selecting the appropriate patients are needed in the future.

Obviously, our study has some limitations. First, some clinical variables or ventilator parameters could not be analyzed due to the retrospective study design. Moreover, this was a single center study with a small sample size, which could not get adequate power to draw definitive conclusions. However, this study included all patients with COVID-19-related AHRF receiving NIV in the city of Shenzhen (with 423 laboratory-confirmed cases on August 31, 2020), and these results could provide some help for the medical staff to select appropriate patients receiving NIV treatment. In future, large-scale prospective randomized control studies are warranted to give us more evidence for the use of NIV and further studies should also explore the treatment effect of different NIV modalities or interface in patients with COVID-19-related AHRF.

In conclusion, close attention should be paid to PaO_2_/FiO_2_ after 1–2 h of NIV initiation in patients with COVID-19-related AHRF receiving NIV. In addition, using NIV as rescue therapy should draw our awareness that it might delay escalation of respiratory support and lead to adverse outcomes.

## Data Availability Statement

The raw data supporting the conclusions of this article will be made available by the authors, without undue reservation.

## Ethics Statement

The studies involving human participants were reviewed and approved by the ethics committee of the Third People's Hospital. Written informed consent for participation was not required for this study in accordance with the national legislation and the institutional requirements.

## Author Contributions

LL, CQ, and RC contributed to determining the outline and content of the study. YF, LG, WW, and SZ contributed to retrieving literature and writing a draft of this manuscript. JY, JW, ZL, and SZ contributed to the data acquisition, the interpretation of outcomes, and data analysis. All authors contributed to revising the draft critically for important intellectual content, providing final confirmation of the revised version, and being responsible for all aspects of the work.

## Conflict of Interest

The authors declare that the research was conducted in the absence of any commercial or financial relationships that could be construed as a potential conflict of interest.
